# Reflections on variability in the blood–breath ratio of ethanol and its importance when evidential breath-alcohol instruments are used in law enforcement

**DOI:** 10.1080/20961790.2020.1780720

**Published:** 2020-08-03

**Authors:** Alan Wayne Jones, Johnny Mack Cowan

**Affiliations:** a Division of Drug Research, Department of Biomedical and Clinical Sciences, Linköping University, Linköping, Sweden; b Department of Public Safety (now retired), Austin, TX, USA

**Keywords:** Forensic sciences, forensic toxicology, blood-ethanol, breath-ethanol, blood–breath ratio, biological variation, drunken driving, law-enforcement, statutory alcohol limits

## Abstract

Variability in the blood–breath ratio (BBR) of alcohol is important, because it relates a measurement of the blood-alcohol concentration (BAC) with the co-existing breath-alcohol concentration (BrAC). The BBR is also used to establish the statutory BrAC limit for driving from the existing statutory BAC limits in different countries. The *in-vivo* BBR depends on a host of analytical, sampling and physiological factors, including subject demographics, time after end of drinking (rising or falling BAC), the nature of the blood draw (whether venous or arterial) and the subject’s breathing pattern prior to exhalation into the breath analyzer. The results from a controlled drinking study involving healthy volunteers (85 men and 15 women) from three ethnic groups (Caucasians, Hispanics and African Americans) were used to evaluate various factors influencing the BBR. Ethanol in breath was determined with a quantitative infrared analyzer (Intoxilyzer 8000) and BAC was determined by headspace gas chromatography (HS-GC). The BAC and BrAC were highly correlated (*r* = 0.948) and the BBR in the post-absorptive state was 2 382 ± 119 (mean ± SD). The BBR did not depend on gender (female: 2 396 ± 101 and male: 2 380 ± 123, *P* > 0.05) nor on racial group (Caucasians 2 398 ± 124, African Americans 2 344 ± 119 and Hispanics 2 364 ± 104, *P* > 0.05). The BBR was lower in subjects with higher breath- and body-temperatures (*P* < 0.05) and it also decreased with longer exhalation times into the breath-analyzer (*P* < 0.001). In the post-absorptive state, none of the 100 subjects had a BBR of less than 2 100:1.

## Introduction

The concentration ratio of ethanol in near simultaneous samples of blood and end-exhaled breath, commonly referred to as the blood–breath ratio (BBR), was originally used to determine a person’s blood-alcohol concentration (BAC) indirectly by the analysis of a sample of breath [1–4]. However, the BBR is a misnomer unless the source of the blood specimen, whether arterial or venous, is specified and the sampling of deep-lung breath is done in a reproducible and standardized way.

Studies have shown that arterial BAC is higher than venous BAC during the absorption phase of the BAC curve, whereas in the post-absorptive state venous BAC exceeds the arterial BAC [5–7]. The venous BBR is therefore expected to vary during absorption, distribution and elimination of alcohol, because the co-existing breath-alcohol concentration (BrAC) runs closer to arterial BAC rather than venous BAC. The concentration of ethanol in end-expired breath is higher than in mixed-expiratory air, because the latter is a mixture of deep-lung and dead-space air [8]. The BrAC is highest after the initial exhalation is rebreathed a number of times prior to sampling, because during rebreathing, ethanol equilibrates between the container (rebreathing bag), the bloodstream and the upper airway mucosa [9–11].

After drinking alcoholic beverages, ethanol is rapidly absorbed into the bloodstream and distributed throughout the total body water compartment. When the blood reaches the pulmonary circulation, any gases and volatiles present will diffuse across the alveolar-capillary membrane at body temperature of ∼37 °C [12]. Because of ethanol’s high solubility in water it is not possible to obtain an end-exhaled sample of breath with the same ethanol concentration as in the alveolar air. There occurs a re-equilibration with the watery mucosa surfaces covering the upper airways and a cooling of the breath from ∼37 °C (alveolar) to ∼34.5 °C as it leaves the mouth [8,13]. Simply on the basis of this 2.5 °C difference in equilibrium temperature, one might expect a 16.3% lower ethanol content in end-exhaled breath compared with alveolar air. This follows because the temperature coefficient of ethanol solubility for blood, plasma and water is 6.5% per 1 °C change in temperature [14].

Another important source of variability in breath-alcohol testing is the subject’s manner of breathing prior to providing a sample; hyperventilation lowers and hypoventilation increases the concentration of ethanol in breath, which impacts on the BBR [15,16]. Unlike respiratory gases, ethanol is much more soluble in water, making it impossible to obtain a breath sample for ethanol analysis that is representative of gas tension in the alveolar regions of the lung [17]. This re-equilibration of ethanol between airway mucosa and the inhaled and exhaled air has led some investigators to question the validity of evidential breath-alcohol testing as a forensic technique for use in traffic-law enforcement [18–20].

Nevertheless, there is no denying the fact that concentrations of ethanol in near simultaneous samples of end-expired breath and in peripheral venous blood are highly correlated over a wide range of BAC and alcohol consumption patterns. Results from controlled drinking experiments and actual drink-driving cases verify the strong association between BAC and BrAC [3,21]. This correlation is highest when comparisons are made during the post-absorptive phase of the BAC curve, which usually commences 30 to 120 min after the end of drinking.

With modern technology for breath-alcohol testing, the accuracy and precision of the measurements are comparable to those obtained when blood samples are analysed at different laboratories using headspace gas chromatography (HS-GC) methods [22,23]. Moreover, the analysis of ethanol in body fluids, including breath, is more reliable evidence that a person exceeded the statutory alcohol limit for driving compared with clinical signs and symptoms of drunkenness and impairment of cognitive and psychomotor functions [24].

This article deals with the concept of a BBR of ethanol and its variability when breath-alcohol instruments are used in law enforcement to test apprehended drivers. The results from a controlled drinking study, involving 100 healthy subjects, were re-evaluated to determine various factors that might influence the BBR [13]. This included their age, height, body weight, body mass index (BMI), gender, ethnicity as well breath- and body-temperature, exhaled volume and time of exhalation into the breath analyzer.

## Methods

### Drinking subjects

One hundred physically fit subjects of both genders (85 men and 15 women) representing three racial groups Caucasians (*n* = 62), Hispanics (*n* = 26) and African Americans (*n* = 12) participated in the study. They were all employees of the Texas Department of Public Safety (Austin, TX, USA). After the study protocol was explained to them all volunteers gave written informed consent to consume a moderate dose of alcohol and provide samples of breath at regular intervals and one sample of venous blood.

At about 1.5 h before drinking started each subject was given one sandwich to eat, so that drinking was not on an empty stomach. The alcohol was administered in the form of whiskey (50.5% v/v) mixed with a carbonated beverage. The dose of ethanol was calculated based on gender and body weight to ensure that BrAC reached at least 0.06 g/210 L by 75–90 min after end of drinking. The diluted whiskey was ingested in three equal portions at 15-min intervals.

### Determination of ethanol in breath

The concentration of ethanol in breath was determined by quantitative infrared spectrometry using an Intoxilyzer 5000 instrument manufactured by CMI Inc. (Owensboro, KY, USA). BrAC of each subject was measured at 15-min intervals until two consecutive results showed a decreasing BrAC, which indicated that the post-absorptive phase of the blood-alcohol curve had been reached. The breath-alcohol analyzer used to determine the BBR was Intoxilyzer 8000, which had been modified by the manufacturer for the purpose of this study. The breath-inlet tube of the instrument was fitted with a thermistor device to record the temperature of breath at the end of an exhalation.

Commencing between 45 and 75 min after end of drinking the subjects were instructed make a moderate inhalation and immediately afterwards a prolonged exhalation into the inlet tube of the Intoxilyzer 8000 instrument. They were instructed to make a forced exhalation for as long as possible at a steady breath flow rate. Two consecutive breath samples were analysed and the mean concentration of ethanol (g/210 L) was later compared with BAC in g/100 mL.

The calibration of the Intoxilyzer 8000 was controlled by analysis of known strength air-ethanol standards generated from a wet-bath simulator operated at 34 °C (Dräger Mark IIA; National Dräger, Houston, TX, USA). An aqueous ethanol stock solution was prepared by taking 78 mL of absolute ethanol (AAPER Chemical, Shelbyville, KY, USA) and diluting this to 1 000 mL with deionized water in a volumetric flask. From this stock solution, 8 mL was diluted to 500 mL with distilled water and transferred to a wet-bath simulator [25]. The Dubowski formula [26] was used to calculate the effluent air-ethanol concentration from the simulator, hence 8 mL of the stock solution after dilution to 500 mL gives an air-vapour concentration of 0.08 g/210 L.

### Determination of ethanol in blood

A specimen of venous blood was taken from each subject immediately after the breath analysis was completed. Blood samples were drawn from a cubital vein using gray-stopper evacuated tubes (6-mL nominal volume) purchased from Beckton, Dickinson (Franklin Lakes, NJ, USA). The blood tubes contained 15 mg sodium fluoride as enzyme inhibitor and 12 mg potassium oxalate as anticoagulant. Prior to making a blood-draw, the skin over a cubital vein was swabbed with antiseptic containing benzalkonium chloride (Professional Disposables, Orangeburg, NY, USA). The tubes containing the blood were inverted 8–10 times to ensure proper mixing with chemical preservatives, then stored in a refrigerator at 4 °C until analysis of ethanol the next day.

The ethanol concentration in blood was determined by HS-GC and a flame ionization detector purchased from Perkin Elmer (Norwalk, CT, USA), model HS 40XL headspace analyzer. This GC instrument was fitted with dual chromatographic columns purchased from Restek (Bellefonte, PA, USA), Rtx-BAC-1 and Rtx-BAC-2 (30 m × 0.32 mm internal diameter).

The HS-GC instrument was calibrated (single point) with an aqueous ethanol standard (0.08 g/100 mL) purchased from Cerilliant (Round Rock, TX, USA), and traceable to NIST. Prior to HS-GC analysis all blood and aqueous ethanol standards were diluted 10 times with n-propanol as the internal standard (0.10 g/100 mL).

All blood samples were analysed in duplicate on the two GC columns thus providing four BAC results from which a mean concentration of ethanol was calculated. The aliquots of blood were taken from two evacuated tubes and two determinations made on each GC column.

### Measurement of body- and breath-temperature

Immediately after the blood samples were taken, the subject’s body temperature was measured in three ways: mouth, tympanic, and forehead. These measurements were made in the same thermostatically controlled room at ∼22 °C in accordance with manufacturers’ instructions. The temperature was measured with a BD Basal Digital oral thermometer (Becton, Dickinson), a Braun ThermoScan IRT 3520 Type 6013 tympanic thermometer (Gillette, Boston, MA, USA), and a TemporalScanner 2000C temporal thermometer (Exergen, Watertown, MA, USA). These devices displayed the temperature measurements digitally and were recorded manually by the instrument operators. The mean of the three observed temperatures was used to represent the subject’s body temperature.

The particular Intoxilyzer 8000 instrument used in the study had been fitted with a thermistor device positioned within the breath-inlet tube and the instrument generated a printed report of the temperature of the breath at end of exhalation.

### Statistical analysis

MedCalc Statistical Software (version 19.1.3), purchased from MedCalc, Ostend, Belgium was used for the statistical analysis. First, a linear regression analysis was done to establish a quantitative relationship between BAC and BrAC for the 100 subjects. Second, a multiple regression analysis was applied to investigate the influence of subject demographics, such as age, gender, BMI, ethnicity, exhaled breath volume, body-temperature, and breath-temperature. In this multivariate analysis, BBR served as dependent variable (*y*) and the other parameters as independent variables (*x*).

A Student’s independent *t*-test was used to compare mean BBR between the sexes and one-way analysis of variance (ANOVA) was used to compare BBR between three racial groups. A Student’s paired *t*-test was used to test whether the mean difference (BAC – BrAC) was significantly different from zero.

## Results

### Descriptive statistics

Demographics of the 100 subjects including their age, height, body weight, and BMI are shown in Table 1. Also shown in the table are mean BAC, BrAC, BBR and the (BAC – BrAC) difference. The variation in breath- and body-temperature, exhalation time into the breath analyzer, and volume of breath exhaled before sampling are also summarized in the table.

**Table 1.** Descriptive statistics for the different variables in 100 subjects and the concentration of alcohol in blood and breath, the blood-breath ratio (BBR) and the difference between breath-alcohol concentration (BAC) and co-existing breath-alcohol concentration (BrAC).

Variable	Mean ± SD	Median	Min and Max
Age, y	29 ± 6	28	21–51
Height, m	1.76 ± 0.09	1.78	1.52–1.96
Body weight, kg	88.0 ± 16.0	89.5	54.0–126.0
BMI, kg/m^2^	28.1 ± 4.4	27.5	18.3–46.2
BAC, g%	0.097 ± 0.015	0.096	0.067–0.147
BrAC, g/210 L	0.086 ± 0.013	0.084	0.061–0.125
BBR	2 382 ± 119	2 366	2 125–2 765
BAC – BrAC, g/210 L	0.0115 ± 0.0051	0.0110	0.0010–0.0260
Breath-temp., °C	34.5 ± 0.4	34.5	33.3–35.5
Body-temp., °C	36.6 ± 0.3	36.7	35.8–37.3
Exhalation time, s	13.7 ± 4.8	12.9	5.9–31.0
Exhaled volume, L	4.14 ± 0.88	4.26	1.50–6.00

**Figure 1.**
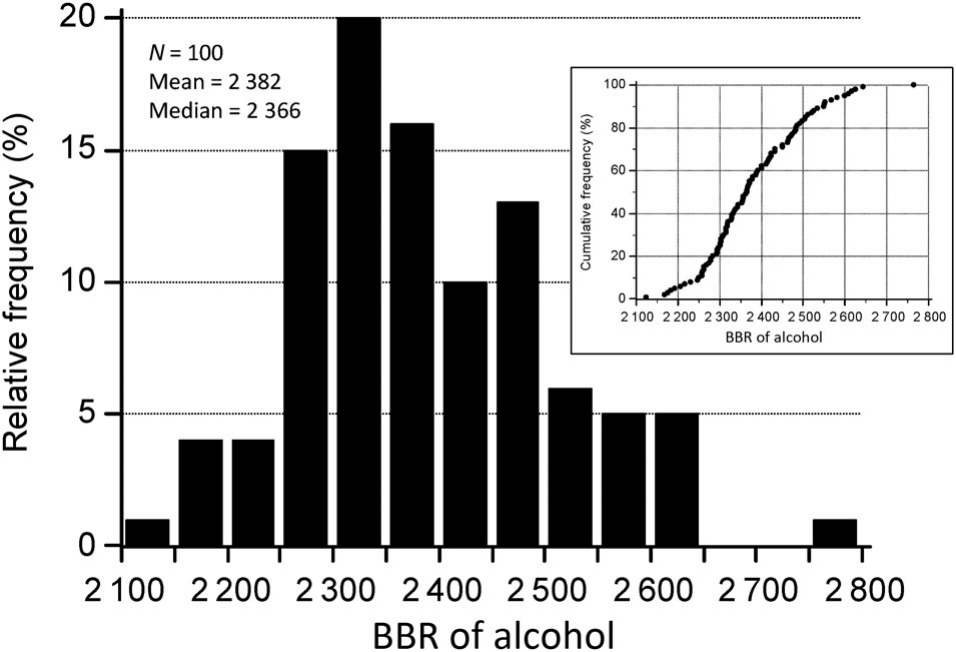
Frequency distribution of venous blood-breath ratios (BBR) of ethanol in 100 subjects who were tested in the post-absorptive phase of the blood-alcohol concentration (BAC) curve. The insert graph illustrates the same data as a cumulative frequency distribution showing no subjects with a BBR less than 2 100.

Figure 1 shows a histogram of individual BBRs and a cumulative frequency plot is shown as an insert graph. None of the subjects had a BBR below 2 100:1 when the breath and blood samples were taken in the verified post-absorptive state.

### Differences between BAC and BrAC

The mean difference between BAC and BrAC was 0.0115 ± 0.0051 (mean ± SD), standard error ±0.00051, which was statistically significant from zero difference (Student’s *t* = 22.5, *P* < 0.001). The smallest and largest differences were 0.0010 and 0.0260, respectively demonstrating that the results from breath-alcohol analysis expressed as g/210 L underestimates BAC in g/100 mL.

### Correlation between BrAC and BAC

**Figure 2.**
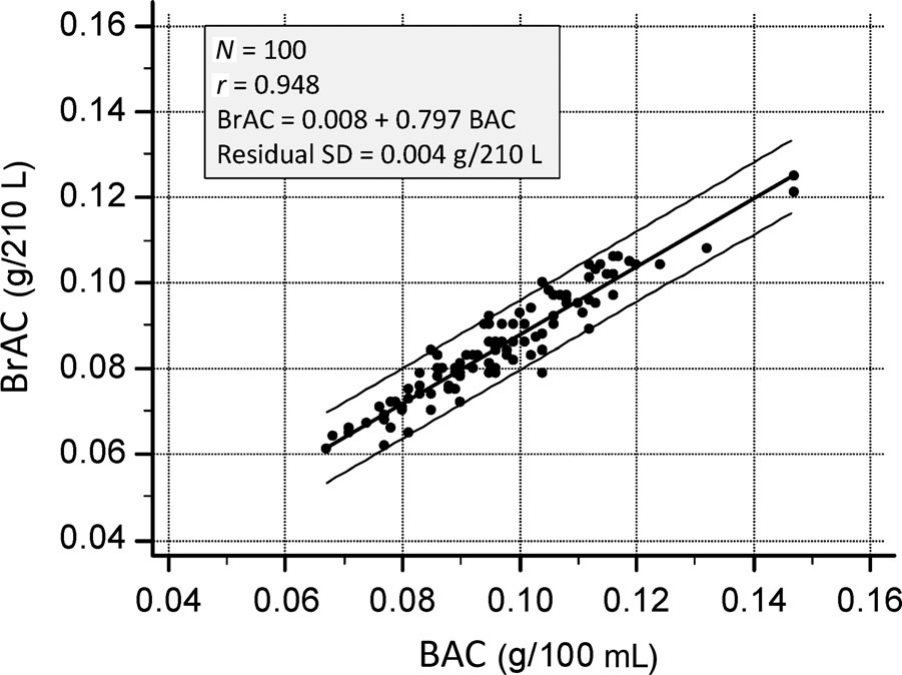
High correlation between venous blood-alcohol concentration (BAC) (*x*-variate) and co-existing breath-alcohol concentration (BrAC) (*y*-variate) when samples were taken during the post-absorptive phase of the BrAC curve. BrAC was determined with an Intoxilyzer 8000 instrument and BAC by headspace gas chromatography.

A scatter plot of venous blood alcohol (*x*-variate) and end-exhaled breath alcohol (*y*-variate) is shown in Figure 2. The Pearson correlation coefficient was statistically highly significant (*r* = 0.948) and the random variations or scatter of points around the regression line (residual SD) was only 0.004 g/210 L, being 8.5% of the mean BrAC.

The regression coefficient (0.797) indicates that as the BAC increases by 0.1 g% the BrAC increases by 0.797 g/210 L, thus a roughly 20% lower concentration on average. This indicates a proportional bias (regression coefficient less than unity) so differences between BAC and BrAC are greater at higher ethanol concentrations.

### Gender and racial differences in BBR

**Figure 3.**
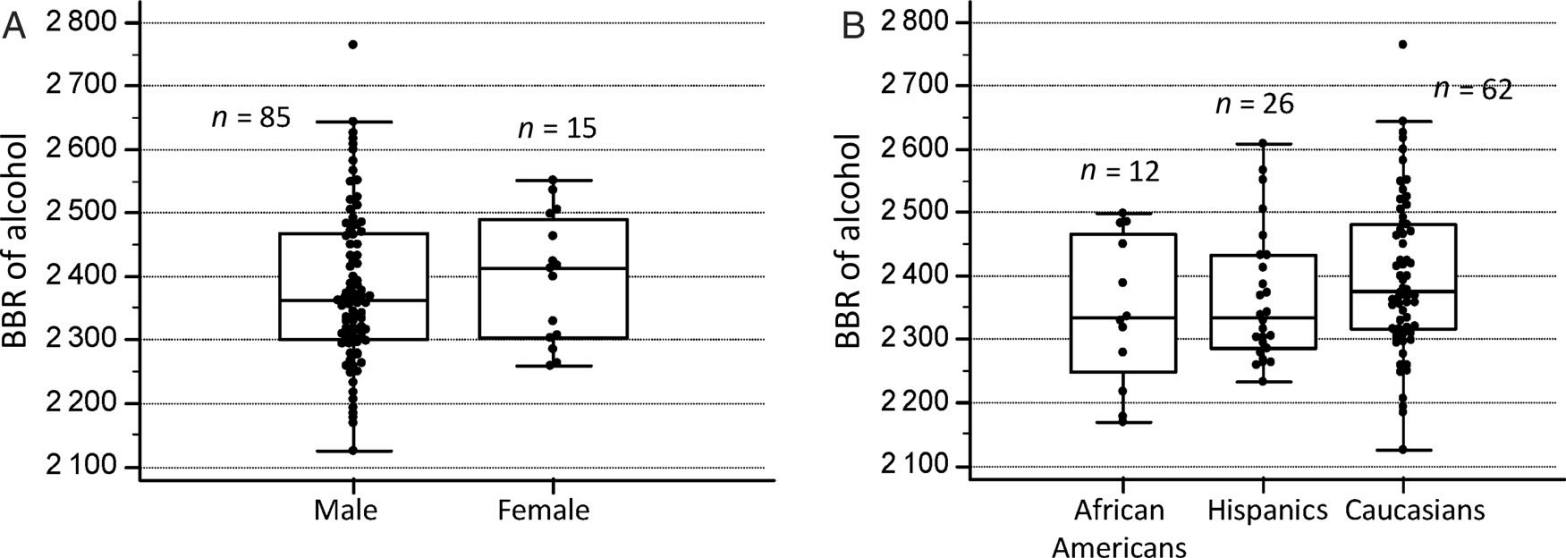
No statistically significant difference between mean blood–breath ratio (BBR) of alcohol for men and women (A) nor between racial groups: Caucasians, African Americans and Hispanics (B).

Figure 3A is a box-and-whiskers graph comparing BBR for male (*n* = 85) and female (*n* = 15) drinking subjects. Although the mean BBR was slightly higher in females (2 396 ± 101) compared with males (2 380 ± 123), the mean difference was not statistically significant according to Student *t*-test (*t* = 0.476, *P* > 0.05) as shown in Table 2.

**Table 2.** Comparison and test of significant difference between mean venous blood/breath ratios of alcohol between different genders and racial groups.

Subgroup	*n*	Mean ± SD	Median	Min and max values
Gender				
Male	85	2 380 ± 123^a^	2 363	2 125, 2 765
Female	15	2 396 ± 101	2 412	2 258, 2 551
Racial group				
Caucasians	62	2 398 ± 124^b^	2 375	2 125, 2 765
Hispanics	26	2 364 ± 104	2 333	2 231, 2 608
African Americans	12	2 344 ± 119	2 333	2 168, 2 498

^a^ No statistically significant difference between means for male and female, *t* = 0.476 (*P* > 0.05).

^b^ No statistically significant difference between means for the three racial groups, *F* = 1.48 (*P* > 0.05).

Figure 3B compares BBRs of alcohol in the three ethnic/racial groups participating in this study. Mean ± SDs were 2 398 ± 124 for Caucasians (*n* = 62), 2 364 ± 104 for Hispanics (*n* = 26) and 2 344 ± 119 for African Americans (*n* = 12) and ANOVA showed no statistically significant difference (*F* = 1.48, *P* = 0.232) as shown in Table 2.

**Table 3.** Differences between co-existing breath-alcohol concentration (BrAC) and venous blood-alcohol concentration (BAC) in relation to a person’s actual blood–breath ratio (BBR), compared with the ratio of 2 100:1 (shaded) used to set the statutory breath-alcohol limit for driving.

Venous BAC g/100 mL	BBR 1 800	BBR 1 900	BBR 2 000	BBR 2 100	BBR 2 200	BBR 2 300	BBR 2 400	BBR 2 500
0.020 0.02	0.023 0.02	0.022 0.02	0.021 0.02	0.020 0.02	0.019 0.01	0.018 0.01	0.017 0.01	0.016 0.01
0.050 0.05	0.058 0.05	0.055 0.05	0.052 0.05	0.050 0.05	0.047 0.04	0.045 0.04	0.043 0.04	0.042 0.04
0.080 0.08	0.093 0.09	0.088 0.08	0.084 0.08	0.080 0.08	0.076 0.07	0.073 0.07	0.070 0.07	0.067 0.06
0.100 0.10	0.116 0.11	0.110 0.11	0.105 0.10	0.100 0.10	0.095 0.09	0.091 0.09	0.087 0.08	0.084 0.08

Results are shown before and after truncation of the third decimal place, which is common practice in many legal jurisdictions.

### Influence of other variables on BBR

**Figure 4.**
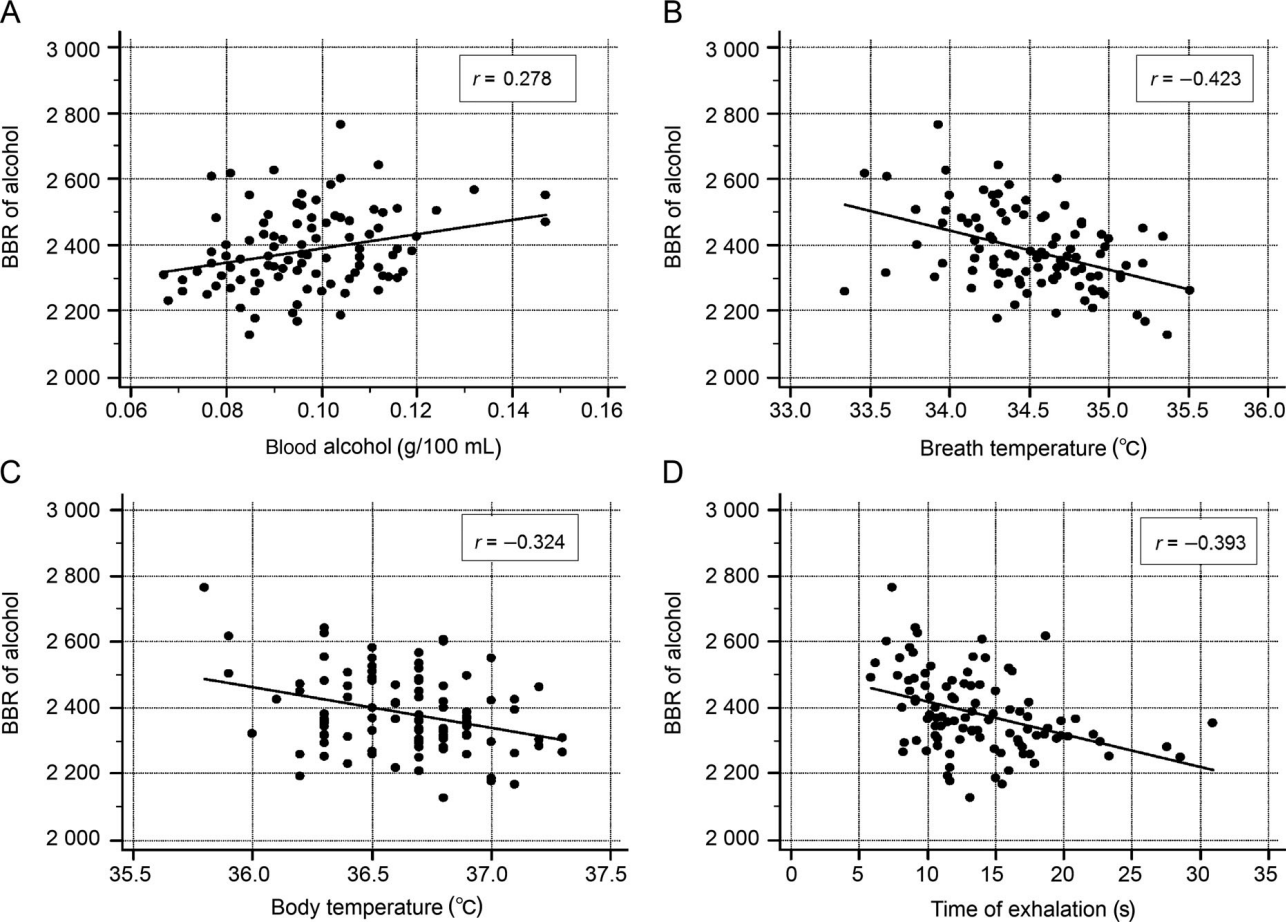
Weak but statistically significant correlations between venous blood–breath ratio (BBR) of alcohol and blood-alcohol concentration (A), end-exhaled breath temperature (B), body-temperature (C), and exhalation time (D) before sampling.

There was only a weak correlation between BBR and age of the test subject (*r* = 0.256, *P* < 0.05), and no statistically significant associations with height (*r* = −0.116), body weight (*r* = 0.045), or body mass index (*r* = 0.119). However, BBR was positively correlated with BAC (*r* = 0.278, *P* < 0.05), and negatively correlated with breath-temperature (*r* = −0.423, *P* < 0.05) and body-temperature (*r* = −0.324, *P* < 0.05). There was also a statistically significant decrease in BBR when subjects exhaled into the breath analyzer for longer times (*r* = −0.393, *P* < 0.05). Scatter plots of these correlations are shown in Figure 4.

### Multiple regression analysis

A multiple regression analysis with BBR as the dependent *y*-variable and seven independent *x*-variables, resulted in a multiple correlation coefficient *R* of 0.659 (*R*^2^ = 0.434, *P* < 0.001). Only four of the independent variables exerted a statistically significantly effect on the BBR and these were the underlying BAC (*P* < 0.05), breath temperature (*P* < 0.01), body-temperature (*P* < 0.05) and the time (duration) of exhalation prior to sampling (*P* < 0.001).

## Discussion

The evidence necessary for prosecution of traffic offenders in most nations requires measuring the concentration of ethanol in a sample of a driver’s blood or breath in close proximity to the time of driving. The sampling and analysis of breath is more practical than having to take blood samples and highly reliable instruments are now available to determine BrAC [27]. In some countries, evidential breath-alcohol instruments are used to estimate the venous BAC and this requires calibration with an assumed population average BBR. However, in most countries, the BBR is used to calculate the statutory BrAC limits from the existing BAC limit. Depending on the choice of BBR, breath-test results might be higher or lower than the co-existing BAC. The BBRs used in various countries to calculate the statutory BrAC are either 2 000:1, 2 100:1, 2 300:1 or 2 400:1, hence there is no international consensus on this question [28].

The results of a controlled drinking study by Cowan et al. [13] were used to determine the influence of various factors on the BBR. These investigators used modern analytical methods to determine ethanol in breath; a multi-wavelength infrared analyzer (Intoxilyzer 8000) for BrAC and HS-GC for BAC. The samples of blood and breath were taken when subjects had entered the post-absorptive phase of the BAC curve as verified by repetitive sampling of breath at 15-min intervals.

In countries like Australia, Canada and the USA, where the Breathalyzer^®^ instrument was used to test driver sobriety, a 2 100 BBR was adopted for legal purposes. This same BBR was used when the statutory BrAC limit for driving was introduced, so a limit of 0.08 g/100 mL in blood became 0.08 g/210 L in breath [29]. However, in reality the BBR varies both between and within subjects depending on many variable factors including subject demographics, state of health and the breathing pattern prior to sampling. Furthermore, the BBR is different for arterial blood compared with venous blood and whether mixed expiratory air or end-expiratory breath was analysed [15,16].

Many blood–breath correlation studies demonstrate that when a 2 100:1 BBR is used for calibration, the venous BAC is underestimated by about 10%–15% provided sampling is done in the post-absorptive phase of the BAC curve [30]. The venous BAC/BrAC is continuously changing during absorption, distribution and elimination stages of the blood-alcohol curve, because arterial-venous differences in ethanol content are also changing. The venous BBR tends to be lower than 2 100:1 during the absorption phase before ethanol is fully equilibrated in all body fluids and tissues [2]. In several studies with apprehended drivers mean BBRs were shown to be closer to 2 300:1 or 2 400:1 rather than 2 100:1 in most cases [3,31,32].

When the UK introduced evidential breath-alcohol instruments in 1983 the government assumed that the population average BBR was 2 300:1 and not 2 100:1 the factor used with the Breathalyzer^®^ instrument [33]. Accordingly, the UK’s existing statutory BAC limit for driving of 80 mg/100 mL led to the creation of a statutory BrAC limit of 35 µg/100 mL as shown below:

BAC limit/BrAC limit = 2 300

BrAC = BAC/2 300 = 80/2 300

BrAC = 0.03478 mg/100 mL or 34.78 µg/100 mL

The BrAC of 34.78 µg/100 mL was then rounded up to 35 µg/100 mL

The creation of a statutory breath-alcohol limit avoids having to convert BrAC into BAC in every case, which has helped to elimination arguments about biological variations in the BBR [34]. However, in order to obtain the best possible sample of breath a test subject is required to make a continuous exhalation for at least 6 s at a certain minimum pressure and flow rate [19,20].

Some people, owing to their age, gender or respiratory dysfunction, might be unable to provide an acceptable breath sample with some evidential breath instruments [35]. When this occurs the best policy is to take a sample of venous blood for analysis instead, which often happens when a driver is injured after a traffic crash. Although accuracy and precision of ethanol determinations in blood and breath contribute to some of the variation in the BBR, the biological and respiratory factors dominate [36].

The data in Table 3 are hypothetical but illustrate the implications of having a BBR different from the assumed value of 2 100:1 when statutory BrAC limits were introduced. The different results from the evidential breath-alcohol tests assume BBRs of between 1 800:1 and 2 500:1. If the person’s BBR was exactly 2 100:1 then numerical BrAC results (g/210 L) will be the same as BAC (g/100 mL) [24,37].

The results in Table 3 indicate that differences between BAC and BrAC are higher when the threshold alcohol limits for driving are 0.08 g/210 L and 0.10 g/210 L compared with 0.02 g/210 L or 0.05 g/210 L [13]. Deviations between BAC and BrAC are less after the third decimal place is truncated, which is customary in many jurisdictions when a suspect is prosecuted. People with actual BBRs greater than 2 100:1 are at an advantage, because their BrAC results (g/210 L) are lower than venous BAC and more likely to be below the punishable limit for driving.

Different jurisdictions use different testing protocols in connection with evidential breath-alcohol analysis, although an important quality assurance requirement is making duplicate tests 2–10 min apart after an initial deprivation period of at least 15 min [38]. The accuracy of the instrument also needs to be controlled by analysis of a known strength air-alcohol standard either generated from a wet-bath simulator or a dry-gas standard [26,39]. This control test should be done either before, after or both before and after a suspect is tested.

Obtaining a good agreement between the duplicate determinations and verification that instrument calibration is within the specifications increases confidence when the results are used in criminal prosecutions. Other safeguards include reporting the lowest of the two breath test results and truncation to two decimals when a suspect is prosecuted. Another approach to give some benefit of the doubt is to make a deduction from the mean analytical result, such as by subtracting 0.015 g/210 L, so that the prosecution BrAC is less than the true value with a high level of certainty.

Some jurisdictions have adopted a guard-band approach to allow for uncertainty. In the UK the statutory BrAC limit for driving is 35 µg/100 mL, but there is no prosecution until a BrAC of 40 µg/100 mL is reached. In countries where the statutory limit for driving is 0.08 g/210 L a prosecution is not initiated unless the result is above 0.09 or 0.10 g/210 L, thus giving an allowance of 0.01 and 0.02 g/210 L, respectively. The enforcement of concentration *per se* statutes and the legal consequences for people convicted of drink-driving offence makes it of great importance for jurisprudence that some allowance is made for analytical uncertainty in the results [40].

BBRs determined in controlled laboratory studies are not necessarily the same as in apprehended drivers for several reasons. Many evidential breath-alcohol testing protocols mandate that the subject makes a continuous exhalation into the instrument for a minimum time of 6 s. Volunteers participating in laboratory studies are more willing to provide a representative specimen of end-exhaled breath, whereas apprehended drivers tend to provide the minimum possible volume of breath to complete the analysis. Furthermore, the BAC reached in laboratory studies ranges from 0.05 to 0.15 g/100 mL, whereas apprehended drivers in most countries have a mean BAC of 0.15–0.18 g/100 mL.

**Figure 5.**
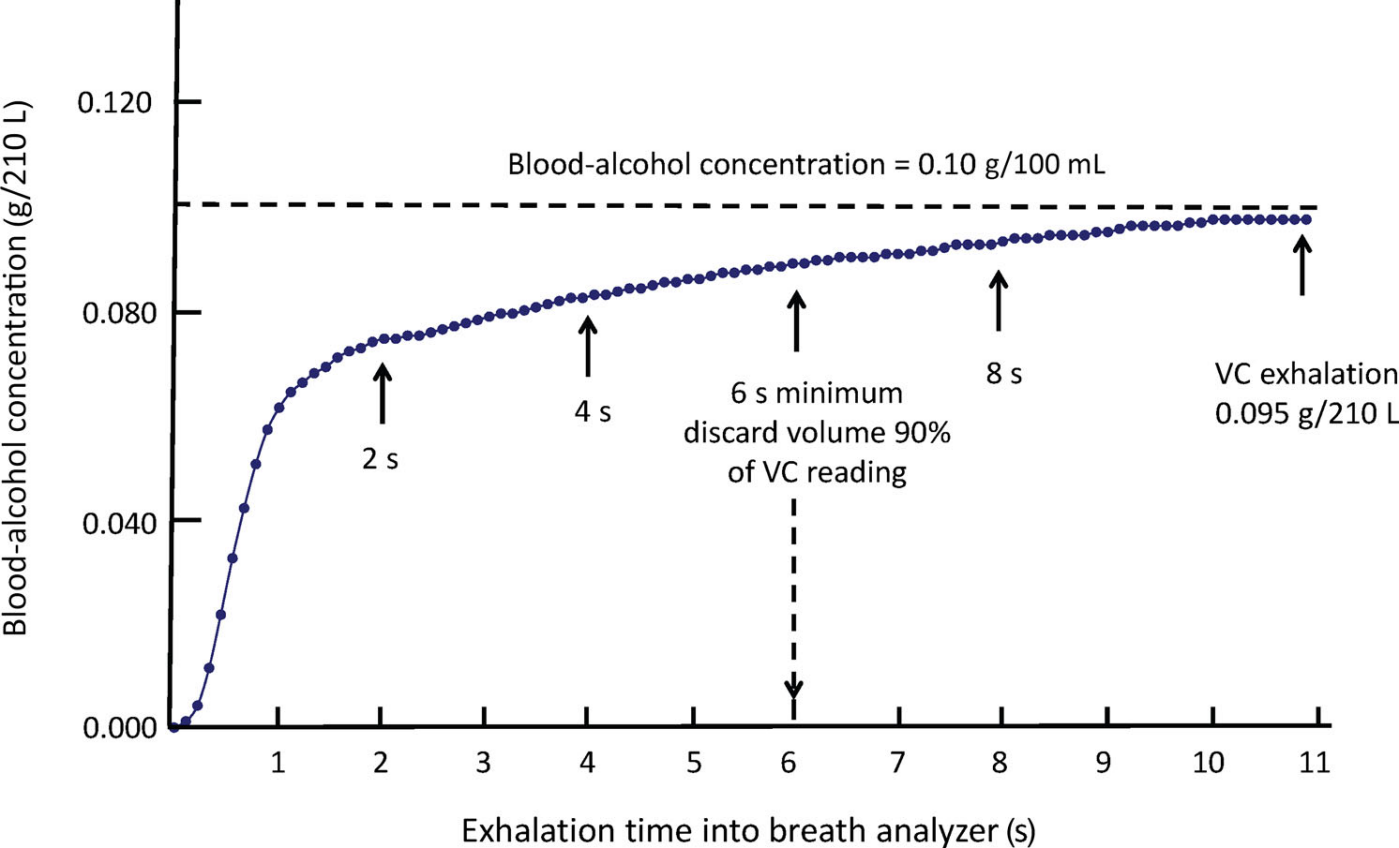
Breath-ethanol profile during a continuous prolonged exhalation into an evidential breath analyzer (Evidenzer). The horizontal line shows the venous blood-alcohol concentration. VC: vital capacity.

Figure 5 shows a typical breath-alcohol exhalation profile when a quantitative infrared analyzer (Evidenzer) was used to test a subject in the post-absorptive phase of the BAC curve. The BrAC increases rapidly after alcohol-free air within the breath inlet tube and the infrared chamber is displaced. The BrAC then increases more slowly as the person exhales past the minimum 6 s requirement and continues to approach a vital capacity exhalation after 11 s. This BrAC trace makes it obvious that if a BBR was calculated after 6 s the result would be higher than after 11 s, which illustrates the importance of obtaining a deep-lung specimen of breath for analysis.

Sampling breath after exactly 6 s (minimum requirement) is obviously an advantage to the suspect compared to an exhalation lasting for 11 s. When evidential breath-alcohol instruments are used in law enforcement, the test person’s BBR is not known precisely, and it might be higher or lower than the assumed 2 100:1 ratio used to set the statutory BrAC limit.

Figure 5 also shows that even after a maximum exhalation for 11 s, the BrAC (g/210 L) was lower than the co-existing venous BAC (g/100 mL). The closeness of agreement between venous BAC and BrAC would have been better if the statutory BrAC limit had been defined as g/230 L rather than g/210 L. The results from the present human dosing study revealed a mean venous BBR of 2 382:1 (median 2 366:1), which would be advantageous to the suspect if a 2 100:1 BBR was used to set the statutory limit for driving. These results are in good agreement with many other studies involving different types of breath-alcohol instrument and experiments in the laboratory and in apprehended drivers [21]. The results of the drinking experiment verified that the mean BBR of alcohol did not depend on gender [41] nor on the person’s ethnicity, whether Caucasian, African American or Hispanic racial groups.

The mean venous BBR would probably have been lower than 2100:1 if testing had been done while ethanol was still being absorbed into the blood stream (rising BAC). Early after drinking ends, the arterial BAC is higher than venous BAC, hence lower BBRs compared with tests done in the post-absorptive state when venous BAC is higher than arterial BAC [42]. Throughout the absorption, distribution and elimination stages of the BAC curve, arterial BAC runs closer to BrAC [5].

The BBR was lower in subjects with higher body- and breath-temperature as might have been expected considering the 6.5% per 1 °C temperature coefficient of solubility resulting in more ethanol entering the air-phase [8,15,43]. The correlation and regression coefficients relating BBR to breath- and body-temperature were negative verifying that values decrease at higher temperatures [14, 44]. BBRs were also lower in subjects exhaling into the instrument for longer times, because as a person reaches his or her vital capacity the BrAC is at its maximum level (Figure 5) [8].

In conclusion, the mean venous BBR in 100 subjects tested in the post-absorptive phase of the BAC curve was 2 382:1 (range 2 125–2 765) with no statistically significant differences between men and women and three racial groups. However, while none of the subjects had a BBR of less than 2 100, there was a trend towards lower values in subjects with higher body- and breath-temperatures and the BBR was also lower when samples were taken after longer exhalation times into the breath analyzer.
